# Effect of two desensitizing agents on dentin permeability *in vitro*

**DOI:** 10.1590/1678-77572016-0228

**Published:** 2017

**Authors:** Hiroshi ISHIHATA, Masafumi KANEHIRA, Werner J. FINGER, Hidekazu TAKAHASHI, Makoto TOMITA, Keiichi SASAKI

**Affiliations:** 1 Tohoku University, Graduate School of Dentistry, Division of Periodontology and Endodontology, Department of Oral Biology, Sendai, Japan.; 2 Tohoku University, Graduate School of Dentistry, Division of Operative Dentistry, Department of Restorative Dentistry, Sendai, Japan.; 3 Tohoku University, Graduate School of Dentistry, Liaison Center for Innovative Dentistry, Sendai, Japan.; 4 Tokyo Medical and Dental University, Graduate School of Medical and Dental Sciences, Oral Biomaterials Engineering, Tokyo, Japan.; 5 Tokyo Medical and Dental University, Clinical Research Center, Tokyo, Japan.; 6 Tohoku University, Graduate School of Dentistry, Division of Advanced Prosthetic Dentistry, Department of Oral Function and Morphology, Sendai, Japan.

**Keywords:** Dentin sensitivity, Dentin permeability, Dentin desensitizing agents, Calcium phosphates

## Abstract

**Objective:**

The aim of this *in vitro* study was to investigate the effect of two desensitizing agents and water on hydraulic conductance in human dentin.

**Material and Methods:**

GLUMA Desensitizer PowerGel (GLU) contains glutaraldehyde (GA) and 2-hydroxyethyl methacrylate (HEMA), and Teethmate Desensitizer (TD) is a powder comprising tetracalcium phosphate (TTCP) and dicalcium phosphate anhydrous (DCPA) that is mixed with water. Deionized water was used as a negative control (CTR). Thirty discs with a thickness of 1.2 mm were cut from the coronal dentin of the third molars and cleaned with 0.5 M EDTA (pH 7.4). After being mounted in a split-chamber device, the discs were pressurized with water at 1 kPa and 3 kPa in order to measure flow rates with a highly sensitive micro-flow sensor and to calculate hydraulic conductance as a baseline value (BL). Following the application of GLU, TD, and CTR (n=10), hydraulic conductance was remeasured with intermittent storage in water after 15 min, 1 d, 1 w, and 1 m. Reduction in permeability (PR%) was calculated from hydraulic conductance. Data were statistically analyzed using nonparametric methods (α<0.05). Representative discs were inspected by SEM.

**Results:**

PR% for GLU and TD were 30-50% 15 min and 1 m after their application. *Post hoc* tests indicated that PR% of CTR was significantly greater than those of GLU and TD at all time points tested. The PR% of GLU and TD were not significantly different. SEM examinations showed noncollapsed collagen meshes at the tubular entrances after GLU, and crystalline precipitates occluding the tubular orifices after TD, whereas CTR specimens showed typical patterns of etched dentin.

**Conclusions:**

The present study on hydraulic conductance in dentin discs treated with two chemically different desensitizing agents and water as a control demonstrated that both products may be characterized as effective.

## Introduction

Dentin hypersensitivity (DH) is a frequently reported pain condition. Depending on whether DH is self-reported or confirmed during a clinical examination, its prevalence varies widely, ranging between 3 – 98%^4^. This variation may be attributed to study samples, the types of practices in which data are collected, and regional variations. Therefore, a careful differential diagnosis is essential because other conditions may produce similar pain. DH is commonly considered a diagnosis of exclusion. The internationally accepted definition of DH, accepted by the Canadian Advisory Board, describes DH as “short, sharp pain arising from exposed dentin in response to stimuli typically thermal, evaporative, tactile, osmotic or chemical and which cannot be ascribed to any other form of dental defect or disease”^3^.

The hydrodynamic theory by Brännström is a globally accepted explanation for DH^1^. Hypersensitive dentin is mostly found in buccal tooth areas, in which enamel is missing because of abrasion, attrition, or erosion. A precondition for DH is that the dentinal tubules should be open at both ends. In short, external stimuli on exposed dentin lead to inward or outward fluid shifts in dentinal tubules that trigger pain due to the stimulation of A-delta nerve fibers around odontoblasts. Based on this theory, a reasonable and logical treatment approach is the occlusion/sealing of peripheral dentin tubules. This concept is widely used to treat DH with an array of different types of desensitizing agents^20^.

Evaluations of dentin desensitizing agents are mostly performed in laboratory studies that predominantly use a human dentin disc model to assess hydraulic conductance, a measure for the ease of fluid flow through dentin prior to and after treatment with desensitizing agents^7,9,10,15,21,23^. Dentin discs may also be used to study morphological details, such as tubular occlusion on the treated surface as well as the partial or total obturation of tubules after fracturing the discs, thereby allowing inspections inside the tubules by SEM or light microscopy^11,18^. *In vitro* testing requires carefully simulated clinical conditions in order to obtain findings that are suitable for predicting clinical behavior with a reasonably high probability. This is particularly important when considering the number of products and methods launched in the market at frequent intervals. *In vitro* findings are often the only documentation available for newly introduced products. However, randomized, placebo-controlled, blinded clinical trials are the gold standard to prove the efficacy of a product or technique. Therefore, the findings of clinical trials are highly desirable as the primary efficacy documentation. Subsequent comparisons with the findings of *in vitro* investigations indicate whether the laboratory set-up used has adequately simulated clinical conditions^12^. However, this procedure is time-consuming and expensive. As a consequence, conflicting and contradicting findings from laboratory tests are often published. These data are not a reliable basis for the qualified prediction of a product’s or a technique’s clinical adequacy.

Recently, the calcium phosphate desensitizer compound Teethmate Desensitizer (TD: Kuraray Noritake Dental Inc., Tokyo, Japan), which is marketed in Japan, North America, and Europe, has gained considerable professional interest because of the product’s high biocompatibility. The main components of TD are tetracalcium phosphate (TTCP: Ca_4_(PO_4_)_2_O) and dicalcium phosphate anhydrous (DCPA: CaHPO_4_), which are eventually transformed in an aqueous environment to hydroxyapatite (HA: Ca_10_(PO_4_)_6_(OH)_2_), the principal mineral in enamel and dentin. This novel product has been evaluated in several *in vitro* investigations^8,11,19,26,27^ and in a randomized, controlled, double-blinded six-month clinical trial, in which it was compared “head-to-head” with GLUMA Desensitizer PowerGel (GLU: Heraeus Kulzer, Hanau, Germany). GLU contains glutaraldehyde (GA)/2-hydroxyethyl methacrylate (HEMA) in aqueous fumed silica dispersion and is used as a positive control to assess equivalency or superiority^17^.

The aim of the present *in vitro* study was to assess hydraulic conductance in two desensitizer agents (TD and GLU) relative to a control at different times (immediate, one day, one week, and one month). The null hypothesis tested was that there was no significant difference among the investigated agents.

## Material and methods

### Specimen preparation

**Figure 1:**
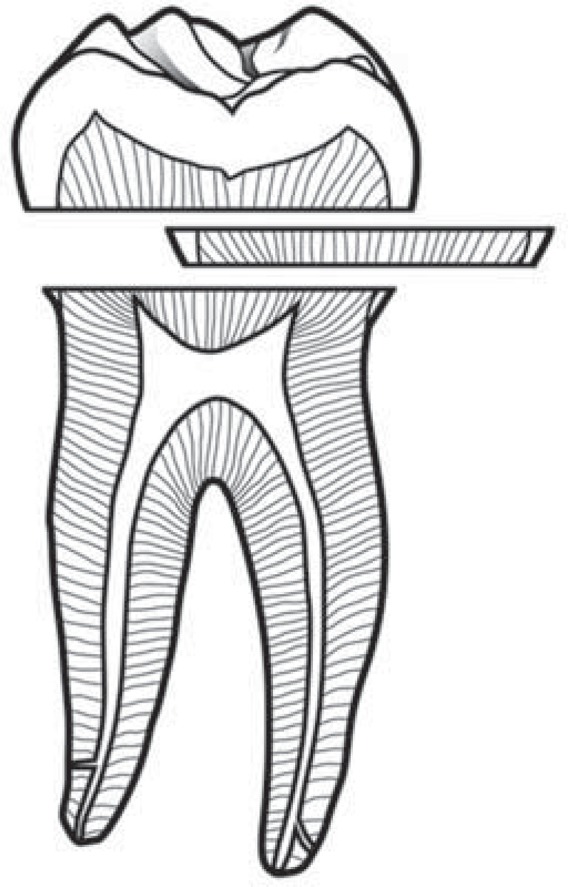
Mid-coronal section of a dentin disc cut perpendicularly to the long axis of a human third molar

Thirty intact human third molars, stored in distillated water immediately after extraction for a maximum of one month, were collected and used with the approval of the Ethics Committee of the Dental Faculty of Tohoku University, Sendai, Japan (Number 26-10, 2015.03.09). Coronal slices with a thickness of 1.2 mm were cut with a diamond wafer saw microtome (Model SP 1600; Leica Microsystems GmbH, Wetzlar, Germany) under water-cooling from mid-coronal dentin perpendicular to the long axis of the teeth. The apical sides of the discs were cut as close as possible to the pulp horns, and the coronal aspects were free from enamel (Figure 1). The discs were rinsed with water, immersed in 0.5 M EDTA solution (pH 7.4), and ultrasonicated for two minutes in order to remove the cutting smear and open the dentinal tubules before a final rinse with deionized water.

**Figure 2:**
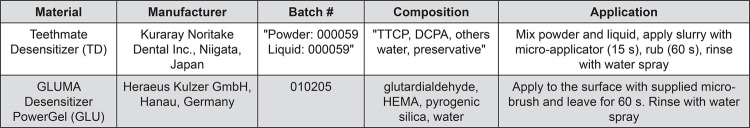
Materials used

TTCP: tetracalcium phosphate; DCPA: dicalcium phosphate anhydrous; HEMA: 2-hydroxyethyl methacrylate

Specimens were randomly allocated to three groups (n=10): GLU application, TD treatment, and water as control agent (CTR). Compositions and applications are listed in Figure 2.

**Figure 3:**
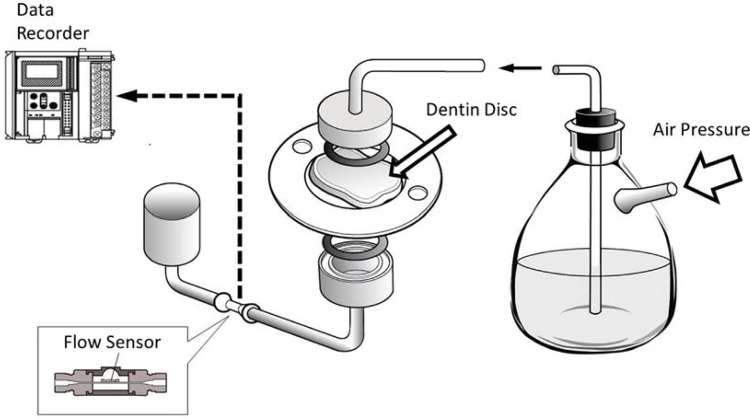
Schematic illustration of the device used to measure the permeability of dentin discs. Water is perfused under adjusted pressure through the dentin sample, clamped between two O-ring sealed chambers, and passes through an electronic micro-flow sensor, in which flow data are registered each 0.1-second intervals and transferred to a data recorder. A target area on the surface of the dentin specimen for the permeation of liquid has been sectioned with a contact sealing circle of O-rings

The schematic drawing in Figure 3 shows the set-up and function of the measuring device. Dentin discs cleaned with EDTA were placed between two O-rings inside a split-chamber device in order to measure hydraulic conductance. Each specimen was mounted on a ring style retainer in which the circumference of the specimen was fixed to the ring with a self-cured resin. The split chamber always accepted the retainer to reproduce an identical position for the measurement area of the specimen. Water was pressurized through the discs from the pulpal side at simulated pulpal pressure of 1 kPa and 3 kPa. The resulting fluid flow/minute was recorded with a microflow sensor (LG16-0150, Sensirion AG, Staefa ZH, Switzerland). Pressure was kept constant for six minutes, and flow was measured at intervals of 0.1 seconds. The system of flow detection took a few minutes from the start of the pressure cycle to obtain a constant flow rate. Therefore, the flow in µL/min was calculated as the mean value of the last four minutes from the cycle and registered on a personal computer. After a three minutes interruption, each of the two subsequent measurement cycles were performed as above. Triplicate measurements of the permeability (filtration) of the specimen were then obtained at the alternative pressure.

After baseline (BL) permeability measurement, specimens were stored in deionized water, mounted in exactly the same position, and remeasured 15 minutes, 1 day, 1 week, and 1 month after the treatment with the respective desensitizing agent or water control using the same procedure described above. In the case of GLU application, the occlusal sides of the discs were first covered with a few droplets of 2% bovine albumin solution (Albumin from Bovine Serum, Cohn Fraction V, pH 5.2; SIGMA ALDRICH, St Louis, MO, USA). On the opposite side of the disc, a vacuum-connected chamber was placed to aspirate the albumin solution into the dentin tubules. After a short rinse of the surface with water, the pretreated specimen was remounted and GLU was applied following the manufacturer’s instructions.

Hydraulic conductance (Lp) was calculated from the mean flow/minute as follows:


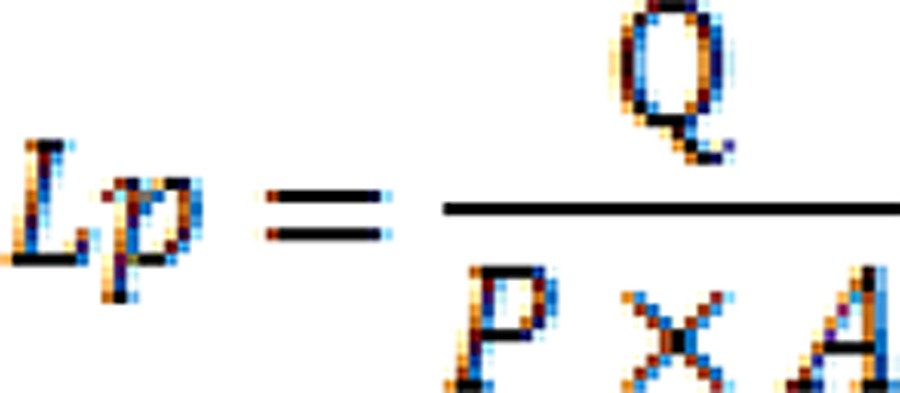


The unit for *Lp* is µL min^-1^cm^-^2^^ cmH_2_O^-^1^^. Q is the flow rate (filtration rate) in µL min^-^1^^, P is the hydrostatic pressure across dentin in cmH_2_O, and A is the surface area in cm^2^ circumscribed by the O-ring.

Lp was calculated at BL as the fiducial value of permeability and at each of the four times after the desensitizer had been applied. In order to normalize the data and be independent from the widely scattering Lp values, the percentage permeability reduction relative to BL (PR%) was calculated at each stage.

### Statistical analyses

The PR% values of the three groups at each time stage were analyzed with the Kruskal-Wallis test and Steel-Dwass *post hoc* test. Difference between PR% at 15 minutes and the other times of each group was evaluated with Wilcoxon Signed Rank test with Bonferroni correction. Calculations were performed using JMP version 11 (SAS Institute Inc., Cary, NC, USA) at a significance level of 5%.

### SEM observation

Following the last determination of the flow rate, three characteristic specimens were selected from each group of discs. After being passed through ascending grades of alcohol, the discs were immersed in hexamethyldisilazane for 10 minutes and then left on filter paper at room temperature for 1 day. Dentin discs were fractured using a dental chisel perpendicular through the surface, coated with Au, and inspected at a 15 kV acceleration voltage on the treated and fractured surfaces (SU-6600, Hitachi High-Technologies Corporation, Tokyo, Japan).

## Results

Table 1 shows the means and standard deviations of hydraulic conductance by material, pressure, and time. BL variations in the three groups were very pronounced. Lp values were markedly larger in the TD group than in the two other groups.

**Table 1:** Hydraulic conductance (Lp) µL min-1 cm-2 cmH2O-1 ; mean (s.d.); n=10

Time Point	kPa	GLU	TD	CTR
BL	1	0.38 (0.19)	0.64 (0.34)	0.28 (0.15)
	3	0.32 (0.18)	0.50 (0.17)	0.24 (0.12)
15 min	1	0.13 (0.04)	0.28 (0.16)	0.27 (0.12)
	3	0.11 (0.05)	0.24 (0.13)	0.23 (0.13)
1 d	1	0.14 (0.07)	0.23 (0.10)	0.27 (0.10)
	3	0.11 (0.06)	0.21 (0.09)	0.22 (0.08)
1 w	1	0.09 (0.06)	0.26 (0.12)	0.27 (0.10)
	3	0.09 (0.05)	0.22 (0.11)	0.20 (0.08)
1 m	1	0.12 (0.07)	0.22 (0.11)	0.26 (0.07)
	3	0.09 (0.06)	0.19 (0.09)	0.20 (0.06)

**Figure 4:**
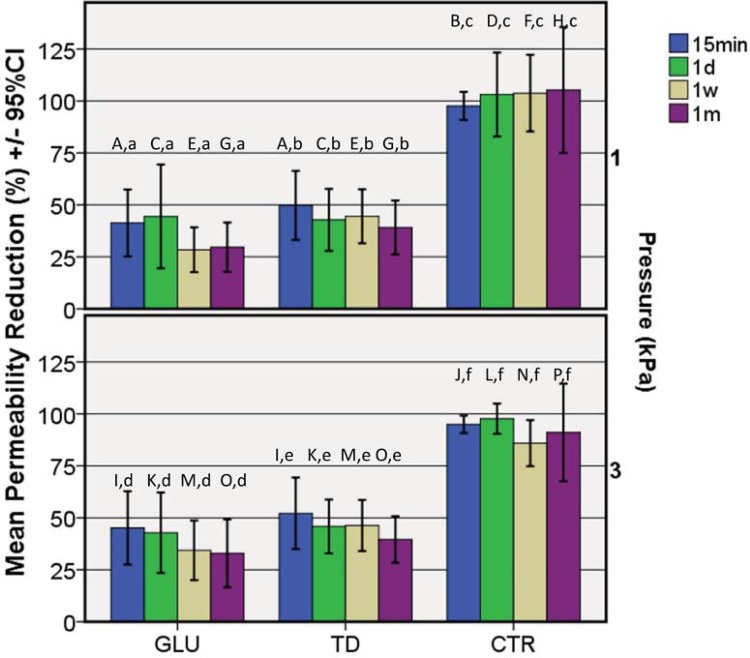
Mean percentage reductions in hydraulic conductance (PR%) and related 95% confidence intervals after one month of storage following two different perfusion pressures of 1 and 3 kPa (n=10). PR% was calculated at each stage after the application of the desensitizing agents and water, respectively, relative to the baseline value measured after EDTA-cleaning (maximum permeability). Bars with the same upper-case letter were not significantly different using Steel-Dwass test. Bars with the same lower-case letter were not significantly different using Wilcoxon Sign Rank test with Bonferroni correction

Figure 4 shows PR% in hydraulic conductance by materials, time, and pressure. The Kruskal-Wallis test suggested that PR% was significantly different at all times regardless of the pulpal pressures used. The Steel-Dwass test indicated that the PR% of CTR was significantly greater than those of the other groups at all times, whereas the PR% of TD and GLU were not significantly different. According to the Wilcoxon Signed Rank Test, no significant change in PR% was observed at the four times until 1 month of post treatment period.

**Figure 5:**
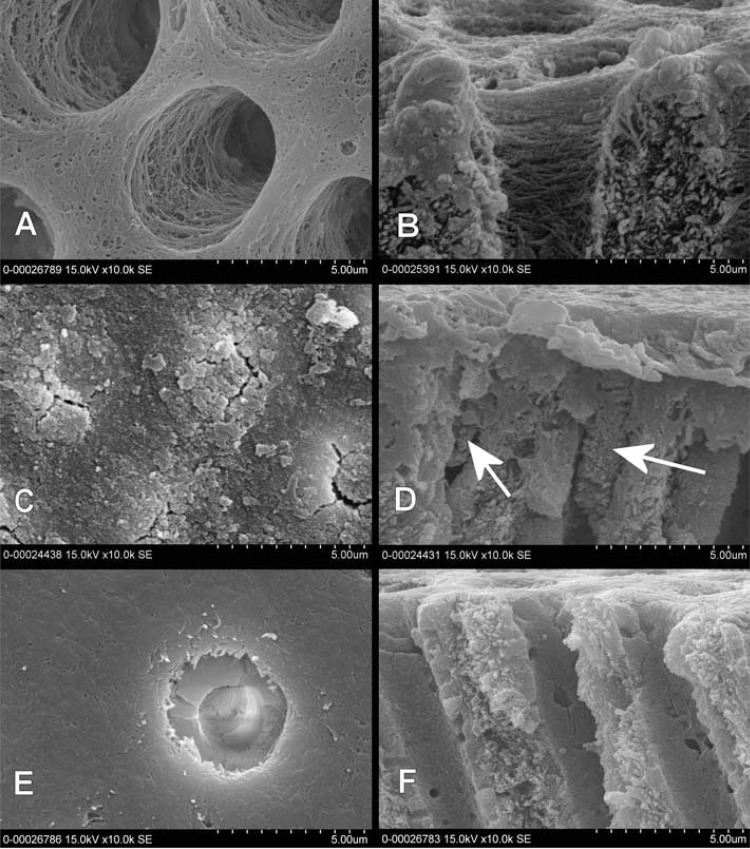
Representative scanning electron microscopy (SEM) micrographs of dentin surfaces and perpendicularly fractured aspects of the same specimens. Regarding GLU (Figures 5A and B), the exposed collagen mesh after EDTA cleaning was not collapsed, but presumably cross-linked by glutaraldehyde with the fibers kept apart. The collagen mesh is observed to a depth of several micrometers. In the case of TD (Figures 5C and D), all tubules are occluded with crystalline precipitates, with similar precipitation being noted on the intertubular surface. Fine precipitates are observed inside the tubules close to the surface (white arrows). The specimen from the CTR group (Figures 5E and F) shows a clean surface and empty tubules, free of precipitates and debris

Figure 5 shows the treated and fractured surfaces of representative disc specimens from each group following the final measurement of the flow rate after one month of storage. SEM photographs 4A and 4B show aspects of free and fractured surfaces after the GLU treatment. Under the EDTA cleaning condition, although peritubular dentin close to the orifice was demineralized, the exposed collagen mesh was not collapsed. Collagen strings were supposedly cross-linked by glutaraldehyde (GA) and kept apart. The collagen mesh was detected to a depth of several micrometers. SEMs C and D show the free surface and a view of the fractured specimen of dentin treated with TD. All tubules and some intertubular dentin were closed and covered with a crystalline grainy substance. The cracks around some of the tubular entrances were considered to be dehydration gaps that had occurred under the high vacuum during sputtering and/or inspection. Very fine-grained crystalline products were observed to a depth of several micrometers (white arrow), and were presumably precipitates from the dissolution process of the primary TD phosphates. The corresponding surfaces of the control specimen after 1-month storage in water are shown in E and F. Free surface and dentinal tubular walls were clean. No remnants of collagen or mineralized precipitates were detected.

## Discussion

The present *in vitro* investigation has provided evidence to support GLU and TD being effective agents for immediate and lasting reductions in dentin disc permeability. Hydraulic conductance after the application of the two commercial products was significantly reduced, whereas water, which was used as a control, had no significant effect throughout the times tested from BL until 1 month. Therefore, the null hypothesis tested in which there was no significant difference among the three agents investigated was rejected.

The active components in the plasma protein precipitant GLU are GA and HEMA. In a spectroscopic investigation, the reaction mechanism between GA and HEMA was described as a two-step reaction. GA reacts with serum albumin to induce precipitation, which mediates the second step of the polymerization of HEMA^24^. Using confocal laser scanning microscopy and scanning and transmission electron microscopy, Schüpbach, et al.^25^ (1997) visualized intrinsically blocked dentinal tubules to a depth of 200 µm inside tubules following the *in vivo* application of GLUMA desensitizer liquid and processing of the extracted teeth. In an *in vitro* study, dentin must be soaked with a protein solution to enable the coagulation reaction, which reduces hydraulic conductance. In the present study, GLU group specimens were soaked in bovine albumin solution, which did not affect the BL permeability of the specimens^13^.

TD is a mildly alkaline product, mainly containing TTCP and DCPA powder that upon mixing with water or an aqueous solution is readily transformed into hydroxyapatite (HA). This transformation is based on a dissolution-precipitation reaction mechanism^6,14,28^. In an aqueous environment, TTCP and DCPA dissolve and supply Ca^2^and PO_4_^3-^. Since this solution is supersaturated concerning apatite, the less soluble compound HA is precipitated. In the oral cavity, new continuously formed HA crystals may be precipitated because of the supersaturation of human saliva with calcium phosphate salts^16^.

The results of the present study showed wide variations in hydraulic conductance values, particularly at BL. Possible reasons for this relatively large scattering may be the age of the donors teeth, the location of the slice cut from coronal dentin, regional variability, and tubule density and diameter. The identification of groups of teeth with uniform conditions as substrates for *in vitro* experiments represents one of the difficulties associated with the permeability testing of dentin.

We selected a very low pressure in order to measure the flow rate through dentin discs, which simulated human pulpal tissue pressure, as reported by Ciucchi, et al.^5^ (1995) and Pashley, et al.^22^(1981). The importance of this variable appears to be greatly underestimated in the literature, and, even in recent studies, pressures of up to 69 kPa have been applied when studying desensitizing agents using a conventional dentin disc model^27^. The main finding of the study conducted almost 20 years ago, in which the authors forced water at five different hydrostatic pressures ranging from 1.3 kPa to 53.3 kPa through dentin slices for 3 hours, was that fluid flow and hydraulic conductance decreased with time in the three middle pressure ranges, but remained constant at 1.3 kPa and 53.3 kPa^2^. The authors concluded that use of high pressure is not recommended because the tubular content appears to be packed against intratubular obstacles. Only a low, physiological pressure is suggested to be suitable for measuring hydraulic conductance in dentin. In contrast to the highly sensitive device used in the present study, the accuracy and resolution of many commonly used instruments may be insufficient, which might explain why other researchers have disregarded this important finding and investigate hydraulic conductance at higher pressures.

In this *in vitro* study, we stored specimens in deionized water to exclude inadvertent mineralization from confounding the results obtained, which is known to occur during storage in artificial saliva that is supersaturated with calcium and phosphate ions. The results obtained for the CTR group confirmed that no significant change in dentin permeability occurred between BL and the end of the 1-month specimen storage in water. Nevertheless, in future studies, experimental methods will be modified using PBS as the liquid to be forced through the discs and artificial saliva as the storage liquid.

In contrast to previous studies, we measured the flow rate at BL from discs that were thoroughly ultrasonicated in neutral EDTA. Thus, free surfaces and at least tubular entrances were cleaned of smear and superficially slightly demineralized. After being soaked in albumin, the exposed collagen will be crosslinked by the aldehyde actions of GLU, and, as observed in SEMs, the collagen surface is noncollapsed. There were no signs of septa bridging the tubules at deeper layers, as reported by Schüpbach, et al.^25^(1997). With TD, the precipitation of HA or precursor phases depends on the presence of nucleation sites for safe bonding to the underlying substrate. Thanatvarakorn, et al.^27^ (2013) reported that the application of TD occluded dentinal tubules and reduced dentin permeability by up to 92% regardless of the exposed collagen network. Consistent with their findings, we achieved excellent immediate permeability reductions in the present study. The SEM of TD-treated dentin surfaces clearly shows the presence of crystallites closing dentinal tubular entrances. However, the fine-grained precipitation of the reaction product was observed inside tubules, and may have been due to the large amount of nucleation points present in peritubular dentin. Due to the supersaturation of saliva with calcium and phosphate in the oral cavity, the ongoing precipitation of HA may occur as a clinically important self-sustaining process.

It was of interest to compare the characteristics of the two products *in vivo* and *in vitro*. In a six-month clinical trial, all patients showed a reduction from 30 to 40% in BL sensitivity for GLU and TD, respectively, both immediately after their application and throughout the six-month duration of the study^17^. Similarly, the present *in vitro* results showed reductions from 30 to 45% in BL and Lp, both early after their application and at the end of the one-month storage period. The immediate decreases in hydraulic conductance after their application and the sustained reductions observed over time were similar to the immediate decrease and sustainability in sensitivity reductions measured clinically on a VAS pain scale. Therefore, the present laboratory test performed under the experimental conditions selected is, to some extent, a predictive tool for the clinical efficacy of desensitizing compounds.

In summary, the present study on hydraulic conductance in dentin discs treated with two chemically different desensitizing agents and in a untreated control demonstrated that both products, according to previous clinical trial data, may be characterized as effective.
